# Trends in organ- and tissue-specific donation refusals in São Paulo, Brazil, a quantitative cross-sectional study

**DOI:** 10.1590/1516-3180.2024.0175.R1.29112024

**Published:** 2025-06-06

**Authors:** Barbara Rossana Gimenez Hidalgo, Rafael Rodrigo da Silva Pimentel, Marcelo José dos Santos, Edvaldo Leal de Moraes

**Affiliations:** I School of Nursing, Universidade de São Paulo (USP), São Paulo (SP), Brazil.; II Postgraduate Program of Nursing Management, School of Nursing, Universidade de São Paulo (USP), São Paulo (SP), Brazil.; III Professional Guidance Department, School of Nursing, Universidade de São Paulo (USP), São Paulo (SP), Brazil.; IV Postgraduate Program in Nursing Management, School of Nursing, Universidade de São Paulo (USP), São Paulo (SP), Brazil.

**Keywords:** Tissue and organ procurement, Nursing, Organ transplantation, Transplants, Brain death, Deceased donors, Organ donation, Tissue donation, Family interview, Family consent, Family refusal

## Abstract

**BACKGROUND:**

The mismatch between the supply and demand for organs and tissues for transplantation is one of the reasons for the high rates of donation refusal. A more recent contributing factor has been the COVID-19 pandemic.

**OBJECTIVES:**

To analyze, through the Terms of Donation of Organs and Tissues, the rates and trends of specific refusals for each organ and tissue from brain-dead donors between 2001 and 2020 in an Organ Procurement Organization.

**DESIGN AND SETTING:**

This was a cross-sectional, exploratory, and retrospective quantitative study on specific donation refusals conducted in one of the ten Organ Procurement Organizations in São Paulo, Brazil.

**METHODS:**

The variables collected included year, age, gender, cause of death, type of hospital, and organs and tissues donated and refused. The data were transferred to Stata software for descriptive and inferential analysis, using generalized linear regression to examine time trends. A significance level of P lt; 0.05 was adopted.

**RESULTS:**

Bones and skin had the highest rates of tissue donation refusal, at 56.40% and 55.37%, respectively. Among solid organs, the pancreas (4.05%) and lungs (5.23%) had the highest refusal rates. In the first decade analyzed, there were increasing time trends in refusals of valves, heart, pancreas, and lungs. In contrast, during the second decade, refusals of valves, kidneys, and pancreas showed decreasing trends. In 2020, the number of refusals for all tissues declined.

**CONCLUSION:**

To reduce tissue-specific refusals, it is important to address and mitigate family beliefs, myths, and negative attitudes toward organ and tissue donation.

## INTRODUCTION

Transplantation is a specific therapeutic option for patients with end-stage organ or tissue failure.^1^ While some organs and tissues can be donated by living donors, most transplants are performed using material from deceased donors.

Countries may adopt different consent policies for organ donation. The most common are the opt-in system, where individuals must formally express their wish to donate, and the opt-out system, in which all individuals are presumed donors unless they have registered otherwise.^2^

In Brazil, the 1997 Transplant Law established the National Transplant System (SNT) to oversee and coordinate transplantation activities. It also created a recipient registry that follows a strict order under the supervision of the Public Prosecution Office and initially implemented an opt-out policy. However, due to a lack of public understanding, the policy was poorly accepted and largely disregarded by healthcare professionals, who continued to seek family consent.^3^ In 1998, a provisional measure abolished the requirement for non-donors to formally object. By 2001, legislation required spousal or family authorization for donation. In 2017, it was clarified that the final decision rests with the legal guardian (spouse, parents, grandparents, children, grandchildren, or siblings) regardless of any verbal or written permission expressed by the potential donor during their lifetime.^4^

Brazil’s organ procurement model combines elements from the North American and Spanish systems. At the national level, the National Center for Notification, Procurement, and Distribution of Organs coordinates efforts with State Transplant Centers, which in turn liaise with municipal authorities through regional Organ Procurement Organizations. This is similar to the U.S. system. At the hospital level, Intra-Hospital Organ and Tissue Donation Committees for Transplantation (CIHDOTTs), modeled after the Spanish approach, operate within each institution.

Organ Procurement Organizations are responsible for coordinating donor identification and logistics within their region, collaborating with medical teams across hospitals and institutions. CIHDOTTs oversee in-hospital donation protocols, maintain death registries, provide continuing education on family support for staff, and manage other aspects of the donation and transplant process.^1^ Trained personnel from both organizations conduct interviews with families of potential donors to determine whether to authorize donation and, if so, which organs and tissues may be retrieved.^5^

Organ and tissue donation is provided free of charge under Brazil’s Unified Health System (SUS), regardless of whether the institution is public or private, as long as the removal is performed by teams and hospitals authorized and supervised by the Ministry of Health, as required by law.^4^

Refusal remains a key factor limiting transplant rates. Reasons for refusal often include cultural or religious beliefs, lack of understanding about brain death, fear of family conflict, hope for a miracle or recovery, uncertainty about the donor’s wishes, discomfort with body manipulation, fear of organ trafficking, mistrust in medical care, inappropriate communication from professionals, and delays in the donation process. ^6-8^

There is a gap in the literature regarding studies that assess refusal rates and trends for specific organs and tissues.

## OBJECTIVE

This study aimed to analyze the rates and trends of specific refusals for each organ and tissue from brain-dead donors between 2001 and 2020, based on the Terms of Donation of Organs and Tissue signed by family members, as recorded by an Organ Procurement Organization in the state of São Paulo, Brazil.

## METHODS

### Design

This was a cross-sectional, exploratory, and retrospective quantitative study on specific refusals of organ and tissue donations from brain-dead donors.

### Setting

The study was conducted at the Organ Procurement Organization of the Hospital das Clínicas of the Medical School of the Universidade de São Paulo (USP), which manages the donation process in a catchment area with a population of 5,979,439 and 96 public and private hospitals.

### Population

The analysis was based on Terms of Donation of Organs and Tissues signed by family members of deceased donors between 2001 and 2020, which indicate the organs and/or tissues authorized or refused for recovery. The database was requested and received in February 2021.

### Data collection

Study variables included: year (2001–2020); donor age (0–11, 12–19, 20–40, 41–59, and over 60 years); sex (female or male); cause of death (cerebrovascular accident, traumatic brain injury, post-cardiopulmonary arrest anoxic encephalopathy, external causes, and others); type of hospital (public or private); and donated and refused organs and tissues (corneas, skin, bones, vessels, valves, heart, liver, kidneys, pancreas, lungs).

### Data analysis

Data were entered into Microsoft Excel (version 16.17) and analyzed using Stata (version 15.0). Initial analysis involved descriptive statistics for sociodemographic, clinical, and institutional variables, as well as the distribution of donations and refusals by organ/tissue and by year (2001–2020).

Time trend analysis was conducted using Prais-Winsten generalized linear regression to estimate β1 coefficients, corrected for first-order autocorrelation, with 95% confidence intervals (CIs). The dependent variable was organ/tissue refusal in the general population and subgroups (male donors, public hospitals, and age groups). Annual Percentage Change (APC) and 95% confidence intervals were calculated using the formula by Antunes and Cardoso (2015): APC = [– 1 + 10 b1]* 100%; 95%CI = [– 1 + 10 b1min.]* 100%; [– 1 + 10 b1max.]* 100%. Relative variation was also calculated as the percentage difference in refusal rates between the first and final years, divided by the initial year’s value.^9^ A 5% significance level was adopted. Trends were interpreted as follows: increasing, positive APC and P lt; 0.05; decreasing, negative APC and P lt; 0.05; and stationary, P gt; 0.05, indicating no significant difference from zero.

### Ethical aspects

This study was approved by the Research Ethics Committee of the Hospital das Clínicas of the Medical School of the Universidade de São Paulo (USP) (Plataforma Brasil opinion n. 4.443.700, issued December 7, 2020), in accordance with international guidelines.

## RESULTS

A total of 2,447 Terms of Donation were analyzed. Most donors were male (1,438; 58.77%), with a mean age of 41.98 years (Standard Deviation: 16.88), ranging from lt; 1 to 82 years. The 41–59 age group represented the highest number of donors (43.24%), and the most common cause of death was stroke (1,254; 51.25%). Over half of the donors (1,361; 55.62%) were from public hospitals (Table 1).

**Table 1:** Characterization of donors (n = 2,447) between 2001 and 2020. São Paulo, Brazil, 2024

Variables	n (%)
**Sex**	
Female	1,009 (41.23)
Male	1,438 (58.77)
**Age group**	
Zero to 11 years old	92 (3.76)
12 to 19 years old	203 (8.30)
20 to 40 years old	718 (29.34)
41 to 59 years old	1,058 (43.24)
60 years old and above	376 (15.37)
**Diagnostic or Cause of Death**	
Brain stroke	1,254 (51.25)
Traumatic Brain Injury	755 (30.85)
Anoxia	158 (6.46)
External causes	176 (7.19)
Others*	104 (4.25)
**Type of institution**	
Public administration	1,361 (55.62)
Private administration	1,086 (44.38)

* Brain abscess, cerebral edema, diffuse cerebral edema, cerebral edema due to diabetic ketoacidosis, cerebral edema due to exogenous intoxication, hepatic encephalopathy, viral encephalitis, hydrocephalus, AVM (arteriovenous malformation), meningoencephalitis, bacterial meningitis, meningococcal meningitis, pneumococcal meningitis, Dandy-Walker syndrome + hydrocephalus, brain tumor, pituitary tumor.

Note: This table characterizes the profiles of the 2,447 potential donors. The variables are: year, donor, sex, cause of death, and type of hospital institution, with the majority being male, in the 41–59-year group, brain stroke as the cause of death, and occurring in public institutions.

A comparison of tissue and organ donations revealed that refusal rates were higher for tissues. Each donor had 5 tissue options (bones, skin, vessels, corneas, valves) and 5 organ options (lungs, pancreas, heart, kidneys, liver) for donation. Over the 20-year period, 12,235 tissues and 12,235 organs were eligible for donation. There were 4,514 refusals for tissues and 321 for organs. Notably, both kidneys and corneas were counted as one pair per donor.

**Figure 1:**
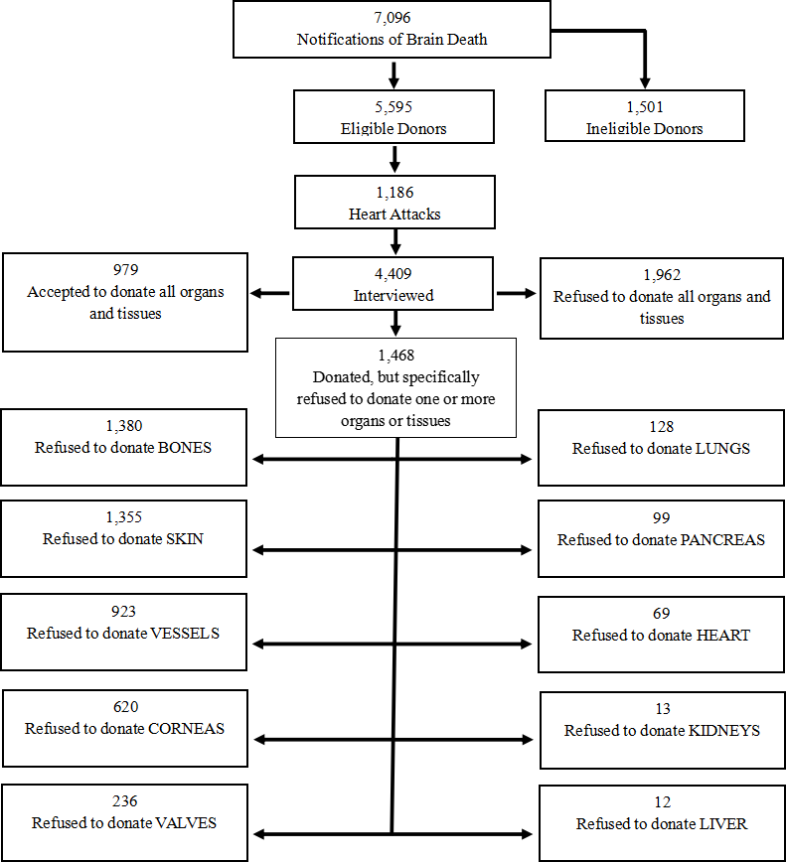
Characterization of notifications, donations and specific refusals of organs and tissues between 2001 and 2020, São Paulo, Brazil, 2024.

Bones and skin were the most frequently refused tissues, with 1,380 (56.40%) and 1,355 (55.37%) refusals, respectively. Among organs, refusal rates were below 6% for all, with the lungs (128; 5.23%) and pancreas (99; 4.05%) having the highest rates (Figure 1).

Brain Death Notifications: notifications sent to the State Transplant Centre of patients with brain death already diagnosed through Resolution of the Federal Council of Medicine number 2.173/2017; Eligible Donors: individuals with brain death already diagnosed without known contraindications; Ineligible Donors: individuals with brain death already diagnosed with known contraindications, such as positive serology (HIV, HTLV, Hepatitis B, Hepatitis C); above the maximum time for withdrawal; with severe infection; with neoplasia; outside the age range; and with other chronic degenerative diseases. Interviewees: interview with family members or people related to the potential donor with the aim of obtaining consent for the donation.

**Figure 2:**
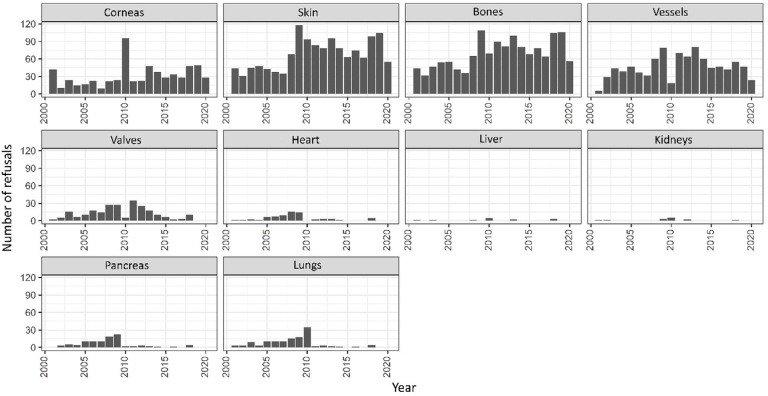
Evolution of specific organ and tissue donation refusals between 2001 and 2020. São Paulo, Brazil, 2024.

From 2009 onwards, refusals for skin, bones, valves, and vessels declined, while cornea refusals rose in 2010. Increases in heart and pancreas refusals were seen in 2007–2008, and refusals for liver, kidneys, and lungs rose in 2010. No organ refusals were recorded in 2019–2020, and tissue refusals also declined during this period (Figure 2).

Between 2001 and 2009, increasing refusal trends were observed for valves (APC = 330.13; 95%CI = 6.41; 1,479, P = 0.009), heart (APC = 65.06; 95%CI = 2.80; 1,121, P = 0.010), pancreas (APC = 268.15; 95%CI = 49.11; 1,444, P lt; 0.001), and lungs (APC = 28.51; 95%CI = 2.38; 256.03, P = 0.007). From 2010 to 2020, refusal rates declined for valves (APC = -0.97; 95%CI = -0.99; -0.58, P = 0.017), kidneys (APC = -0.27; 95%CI = -0.46; -0.14, P = 0.040), and pancreas (APC = -0.29; 95%CI = -0.49; -0.01; P = 0.038) (Table 2).

**Table 2:** Time trend of the percentage of organ and specific tissue donation refusals in two periods: 2001-2009 and 2010-2020. São Paulo, Brazil, 2024

Organ/Tissue	2001 n (%)	2009 n (%)	APC (95%CI)	Δ%	P value	2010 n (%)	2020 n (%)	APC (95%CI)	Δ%	P value
Corneas	42 (70.00)	23 (13.29)	-0.99 (-1.11; 0.28)	-81.01	0.057	96 (57.49)	28 (18.54)	-0.86 (-0.99; 18.05)	-67.75	0.387
Skin	44 (73.33)	118 (68.21)	13.79 (-0.90; 23.97)	-6.98	0.248	93 (55.69)	55 (36.42)	-0.90 (-0.99; 4.01)	-34.60	0.215
Bones	44 (73.33)	109 (63.01)	3.57 (-0.97; 75.57)	-14.07	0.499	69 (41.32)	56 (37.09)	-0.66 (-0.99; 102.31)	-10.23	0.569
Vessels	5 (8.33)	79 (45.66)	204.07 (-0.92; 575.43)	448.13	0.121	18 (10.78)	23 (15.23)	-0.96 (-0.99; 11.88)	41.28	0.228
Valves	2 (3.33)	27 (15.61)	330.13 (6.41; 1.479)	368.76	0.009	5 (2.99)	0 (0)	-0.97 (-0.99; -0.58)	-	0.017
Heart	1 (1.67)	14 (8.09)	65.06 (2.80; 1.121)	384.43	0.010	0 (0)	0 (0)	-	-	-^†^
Liver	1 (1.67)	0 (0)	-0.14 (-0.39; 0.15)	-100	0.242	4 (2.40)	0 (0)	-0.12 (-0.39; 0.20)	-	0.356
Kidneys	1 (1.67)	3 (1.73)	-0.08 (-0.59; 0.94)	3.59	0.755	5 (2.99)	0 (0)	-0.27 (-0.46; -0.14)	-	0.040
Pancreas	0 (0)	23 (13.29)	268.15 (49.11; 1.444)	-	< 0.001	2 (1.20)	0 (0)	-0.29 (-0.49; -0.01)	-	0.038
Lungs	3 (5.00)	17 (9.83)	28.51 (2.38; 256.03)	96.6	0.007	35 (20.96)	0 (0)	-0.89 (-0.99; 0.28)	-	0.074

This table shows the time trend of the percentage of specific organ and tissue donation refusals from to periods: 2001-2009 and 2010-2020. In the period 2001-2009, there was an increasing trend to refuse to donate valves, hearts, pancreas, and lungs, and a stationary trend for other organs and tissues. In the 2010-2020 period, refusals for valves, kidneys, and pancreas tended to decrease, whereas those for other organs and tissues showed stable trends.

APC = Annual Percentage Change; CI = confidence interval.

Refusal trends remained stationary over both decades for corneas, skin, bones, vessels, and liver. Kidney refusal rates were stationary from 2001 to 2009, as were lung refusal rates from 2010 to 2020.

## DISCUSSION

The analysis of the results shows a decrease over the years in the number of specific refusals to donate organs and tissues for transplantation, as recorded in the Terms of Donation signed by family members. Although this result is encouraging, it is important to note that refusal rates for tissues remain higher than for organs. This discrepancy may be associated with the greater social visibility of organ donation compared to tissues, whose therapeutic use is less familiar to the public.^10^

Media coverage has played a significant role in shaping public opinion on organ donation in recent decades. Brazilian soap operas, news reports, and advertising campaigns have influenced decisions to donate.^11^ With their broad reach, soap operas are able to construct long-running narratives that integrate real information about organ donation with dramatic storytelling, generating sustained public engagement. Between 1992 and 2018, six Brazilian soap operas addressed the theme of adult and pediatric transplantation.^11^

National news reports on transplantation, whether negative or positive, also have a strong impact, especially when combined with soap opera storylines. A notable example occurred in September 2013, when socialite Chiquinho Scarpa buried his Rolls-Royce as part of a campaign titled: “It’s absurd to bury something much more valuable than a Bentley: your organs.”^11^ This campaign was followed by a decrease in refusal rates for heart, kidney, pancreas, and lung donations that same year.

Publicized incidents involving deaths and subsequent donations have also influenced public attitudes. In 2008, the case of 15-year-old Eloá Cristina Pimentel, who was held captive for 100 hours and fatally shot by her ex-boyfriend, led to a notable response when her mother authorized organ donation. At least six individuals benefited, and the case received considerable attention, correlating with a drop in refusal rates in 2008 and 2009.^11^ Similarly, the 2011 school massacre in Rio de Janeiro, where families consented to organ donation, had a positive public impact—although refusal rates still increased in 2011 and 2012.^11^

Since 2013, the Ministry of Health has conducted the annual “Green September” campaign, producing videos, posters, brochures, and other materials targeted at healthcare professionals and the general public. Topics have included notifying families (2014), inspiring stories of transplant-recipient athletes (2015), and testimonials from recipient and donor families (2019). However, the campaigns appear to have limited impact on refusal rates, likely due to deep-rooted mistrust in the public health system and enduring myths.^11^

Tissue donations, such as skin and bone, have higher refusal rates, which may be linked to a lack of information and concerns among family members about the donor’s post-mortem appearance.^12,13^ In addition, media campaigns tend to focus more on organ donation, further contributing to this disparity.

In contemporary society, physical appearance holds significant social value, which may lead family members to decline skin donation to preserve the donor’s appearance. A study in Saudi Arabia found that individuals with lower education levels were more likely to oppose skin donation.^14^ This barrier can limit access to accurate information and hinder discussions on the topic.

TV series, movies, and soap operas often perpetuate misconceptions about skin donation. Decision-making may be compromised when skin removal is equated with the skinning of animals, a perception reinforced by comparisons between surgeons and butchers.^13^

The reduction in skin donation refusal in 2014 may be associated with the 2013 fire at the Kiss Nightclub in Santa Maria in the southern state of Rio Grande do Sul,^15^ which resulted in a shortage of skin grafts for victims. This tragedy may have raised public awareness and sensitivity toward skin donation.

The high refusal rate for bone tissue found in this study may stem from concerns about bodily disfigurement, lack of clear communication, and inadequate or absent requests for bone donation by healthcare professionals. This may be due to a general lack of societal awareness regarding the possibility of bone tissue transplantation.^12^

A Brazilian study found that 62.7% of families approached for organ donation were not asked about bone donation. Among the legal guardians asked about the donation of bone tissue, 92.9% did not receive information about which bones would be removed, and 96.5% were not told how the donor’s body would be reconstructed. Furthermore, 60.7% of families declined bone donation to preserve the donor’s appearance, and 39.3% cited lack of information about the procedure.^16^

These findings underscore the need for measures to reverse this situation, as bone transplantation is essential in cases of accidents or serious diseases that involve the restoration of this tissue.^17^ Awareness campaigns remain heavily focused on organ donation, with little emphasis on tissue donation, such as skin and bone. A shift in campaign content and media narratives is essential to educate the public on the types of organs and tissues that can be donated, the donation and graft removal processes, and the uses of donated tissues.

Throughout the study period, organ donation consistently showed lower refusal rates compared to tissues. This trend is mirrored internationally. In the United Kingdom, a 2020 study reported refusal rates of 3.38% for the heart and 2.6% for the lungs.^18^ In Australia and New Zealand, the refusal rates for common organs were even lower—0% for the liver, 1.35% for the lung, 1.89% for the pancreas, and 3.81% for the heart. In contrast, refusal rates were higher for less commonly transplanted tissues: heart valves (8.55%), stomach and intestines (12.82%), corneas (32.17%), and bones (51.95%).^19^

In addition to the data available in donation registries, public opinion surveys offer insights into specific refusals. A 2018 German study found that while 72% of respondents were willing to donate, 13% would refuse specific organs or tissues. Of those who would refuse, the specific refusal rate was 56% for the cornea, 27% for the heart, and 11% for the skin.^20^ In a United States survey, 49% of teenagers expressed willingness to donate their organs after death. Of these, 33% refused to donate the following organs and tissues: eyes (32%), pancreas (13.8%), lungs (12.8%), and heart (9.9%).^21^

These findings suggest that individual preferences regarding donation are more nuanced than a simple “donor” or “non-donor/refusal” status. National donation registries, national and regional surveys, and social science studies reveal that willingness to donate varies by tissue type, with a higher likelihood of donating vital organs such as kidneys, lungs, livers, and hearts, and less willingness to donate tissues such as skin, bones, valves, vessels, eyes, and corneas.^18^

Although refusal rates for the pancreas and lungs are relatively low, they are still higher than for other organs, suggesting the need for further investigation. It is hypothesized that the donor’s medical history, such as diabetes for the pancreas and smoking for the lungs, may influence refusal decisions. In addition, a study in China found that religious and cultural beliefs may deter donation of lungs and hearts, as these organs are traditionally considered the seat of the soul.^22^

Trend analysis reveals that between 2001 and 2009, refusals for the pancreas, heart, and lungs increased. These refusals may have been influenced by the interviewers’ judgments about prior pathologies that would render the organs unusable. In the case of the heart, its symbolic and emotional significance may have contributed to refusal.^22^

In contrast, from 2010 to 2020, refusal rates for the pancreas and kidneys declined, and the rates for other organs remained stable. This shift may reflect the implementation of a law requiring hospitals within the Organ Procurement Organization’ jurisdiction to assign qualified transplant coordinators.^23^ The establishment of the National Day of Organ Donation^24^ and its media promotion may have also contributed to the trend reversal.

Valve refusal significantly increased from 2001 to 2009, possibly due to a lack of trained tissue recovery teams, which discouraged interviewers from requesting valve donation. In contrast, in the period from 2010 to 2020, refusal rates declined, potentially due to the reactivation of tissue recovery teams, which may have encouraged interviewers to request tissue donation.

The family interview plays a crucial role in the donation process. Beyond the interviewer’s approach, factors such an appropriate interview setting, transparent communication, quality care for the potential donor and their relatives during the hospitalization period, and adherence to ethical and legal standards can all affect the decision. Interviewers must use appropriate language, understand the donor’s and family’s background, empathize with their emotional state, and be trained in the science and ethics of donation.^25^

The Terms of Donation, the sole document authorizing organ and tissue donation, may differ slightly by state. In 2010, the form was restructured to specify refusals instead of listing accepted organs and tissues, thereby making the process more specific. This change may explain the decrease in refusal rates for most tissues in 2010, except for corneas, as shown in a previous study.^26^

It is noteworthy that refusal rates for corneas, skin, bones, and vessels remained unchanged across both decades studied. These data suggest that no measures were taken to change the situation or that the actions were ineffective, reinforcing the need for targeted education and training efforts.^27^

In 2020, none of the families who agreed to donate refused the recovery of any organ. This may be partly attributed to the media coverage surrounding the organ and tissue donation of Gugu Liberato, a very popular Brazilian TV presenter who died in November 2019.^11^ Following this, Google Trends data showed a spike in searches related to “Organ and Tissue Donation” and “Brain Death” across Brazil.

Despite the insights gained, this study has limitations regarding the external validity of the findings and should be replicated in other contexts. In addition, the Terms of Organ and Tissue Donation used in data collection do not include fields for specifying reasons behind individual refusals. Therefore, further qualitative research is necessary to explore these motivations, particularly those related to family characteristics.

Finally, this study highlights the importance of analyzing specific organ and tissue refusals, as well as the need to identify the reasons for refusal. It recommends that the Donation Authorization Form be updated to include a field for documenting reasons for each refusal. Moreover, family interviews should include clear information about the importance and feasibility of tissue donation, alongside organ donation.

## CONCLUSION

The data analysis indicates that refusals to donate organs and tissues have decreased over the years. However, non-consent rates for certain tissues remain high. This discrepancy warrants further investigation through targeted studies to better understand the underlying causes, which may be linked to public policies that prioritize organ donation while placing limited emphasis on the importance of tissue donation.
